# Why Antagonistic Traits against Cytoplasmic Incompatibility Are So Elusive

**DOI:** 10.3389/fmicb.2016.00392

**Published:** 2016-03-31

**Authors:** Ranjit Kumar Sahoo

**Affiliations:** School of Biology, Indian Institute of Science Education and Research ThiruvananthapuramThiruvananthapuram, India

**Keywords:** *Wolbachia*, *Cardinium*, sex ratio, cytoplasmic incompatibility, antagonistic evolution, population, maternal inheritance, intracellular symbiont

## Introduction

Intracellular symbionts are present in a wide variety of animals, plants and other life forms (Correa and Ballard, 2016); and have an intriguing lifestyle. To enhance their spread, intracellular symbionts have evolved a variety of mechanisms. Some of them provide benefits to their host and are effectively mutualists (e.g., *Buchnera* in aphids; Douglas, 1998), while others bias the sex ratio of the hosts' offspring toward females (e.g., *Wolbachia* in many insects; Reuter et al., 2008; Werren et al., 2008; Zug and Hammerstein, 2015). In the latter, the mode of transmission is strictly maternal. Therefore, such symbionts induce their female hosts to produce higher numbers of daughters than the average number produced in the population (Reuter et al., 2008). A more indirect strategy impedes reproduction of uninfected females, where infected males that mate with uninfected females render the female sterile by cytoplasmic incompatibility (CI; Werren et al., 2008).

CI is a type of conditional sterility independently evolved in two bacteria: *Wolbachia* (Alphaproteobacteria) and *Cardinium* (Bacteroidetes) that infect arthropod hosts (Penz et al., 2012; Santos-Garcia et al., 2014). CI occurs when a host gamete from an infected male fuses with the gamete of either an uninfected female or that of a female infected with a different strain typically resulting embryonic death (Tram and Sullivan, 2002; Werren et al., 2008; Correa and Ballard, 2016). As a consequence, the infection frequency increases in the population due to reduced fitness of uninfected females instead of any direct benefit to infected females (Champion de Crespigny et al., 2005). Such costs in fitness led many authors to speculate that antagonistic strategies could have evolved in the host against the symbiont (Turelli, 1994; Charlat et al., 2003; Vala et al., 2004; Crespigny and Wedell, 2007; Champion de Crespigny et al., 2008), although most of the studies have failed to confirm antagonisms that evolved exclusively in response to CI-phenotype (Vala et al., 2004; Crespigny and Wedell, 2007). Antagonistic strategies include any strategy against the CI-phenotype through either avoidance or resistance mechanisms. Moreover, when the evolution of such strategies was modeled, the results were varied; showing that evolution of CI-antagonism is conditional, for instance, it could depend on the frequency of CI in the population (see Champion de Crespigny et al., 2005, 2008). Therefore, it remains unclear whether the evolution of such strategies is possible. Hereby, I provide a conceptual framework taking in consideration that infection frequency affects the proportion of a population under selection for CI-antagonism; this may explain why CI-antagonistic traits have been so elusive.

## The conceptual framework

In a population under selection, a new variation tends to persist when the selective benefit outweighs the associated cost. Persistence of such beneficial variation depends on the (i) number of individuals under selection and its temporal fluctuation, because reduced number of individuals decrease the chance that such a variation would be available among them and increase the role of drift that can potentially eliminate the same, and (ii) dynamics between the cost and benefit of the variation as one can outweigh other based on population structure. Therefore, when evaluating the fate of a potential variation under selection, it is necessary to simultaneously consider the temporal dynamics of population structure and the effect of the variation.

Since the standard model of evolution assumes that a selection pressure equally affects all individuals of a population, a steady number of individuals is always available to selection. However, such a model may not apply to the population which is under selection due to CI-inducing symbiont. I argue that as the number of individuals—who potentially experience the fitness cost due to CI-phenotype—fluctuates over time, the strength of selection changes; and this, in turn, makes the evolution of any antagonistic strategies against CI-phenotype unlikely. In the case of traits against CI-phenotype, this number is determined by the frequency of infection in the population. When the number of individuals under selection is low, there is less variation (if any) for natural selection to act on, and genetic drift will play a larger role so that adaptive genes have a high risk of extinction.

To show how CI divides a population and thus reduces the likelihood of evolution of traits against CI-phenotype, I categorized a *Wolbachia*-infected host population into four classes based on their sex and infection state. Then, I investigated the plausibility of the evolution of antagonistic strategies against CI-phenotype if any, in each category at different levels of infection prevalence. I have also derived alternate questions to understand the dynamics of CI-phenotype at intermediate infection frequency.

### Assumptions and attributes

I have assumed a single strain *Wolbachia* incidence in the host population, its perfect vertical transmission and complete effect of CI. I have also assumed for no horizontal transmission within the population and no multiple incidences—hence uninfected individuals are not susceptible to infection. It may be good to note that infection rates in males and females are always the same, and the sex ratio of the population remain 1:1.

Thus, in a population infected with *Wolbachia*, gametes from infected male unable to successfully fertilize uninfected eggs, which causes a (i) reduction in fecundity of uninfected females, (ii) relatively higher reproductive success of infected females that transmits *Wolbachia* to next generation, and (iii) infection spreads through the population.

**Figure 1:**
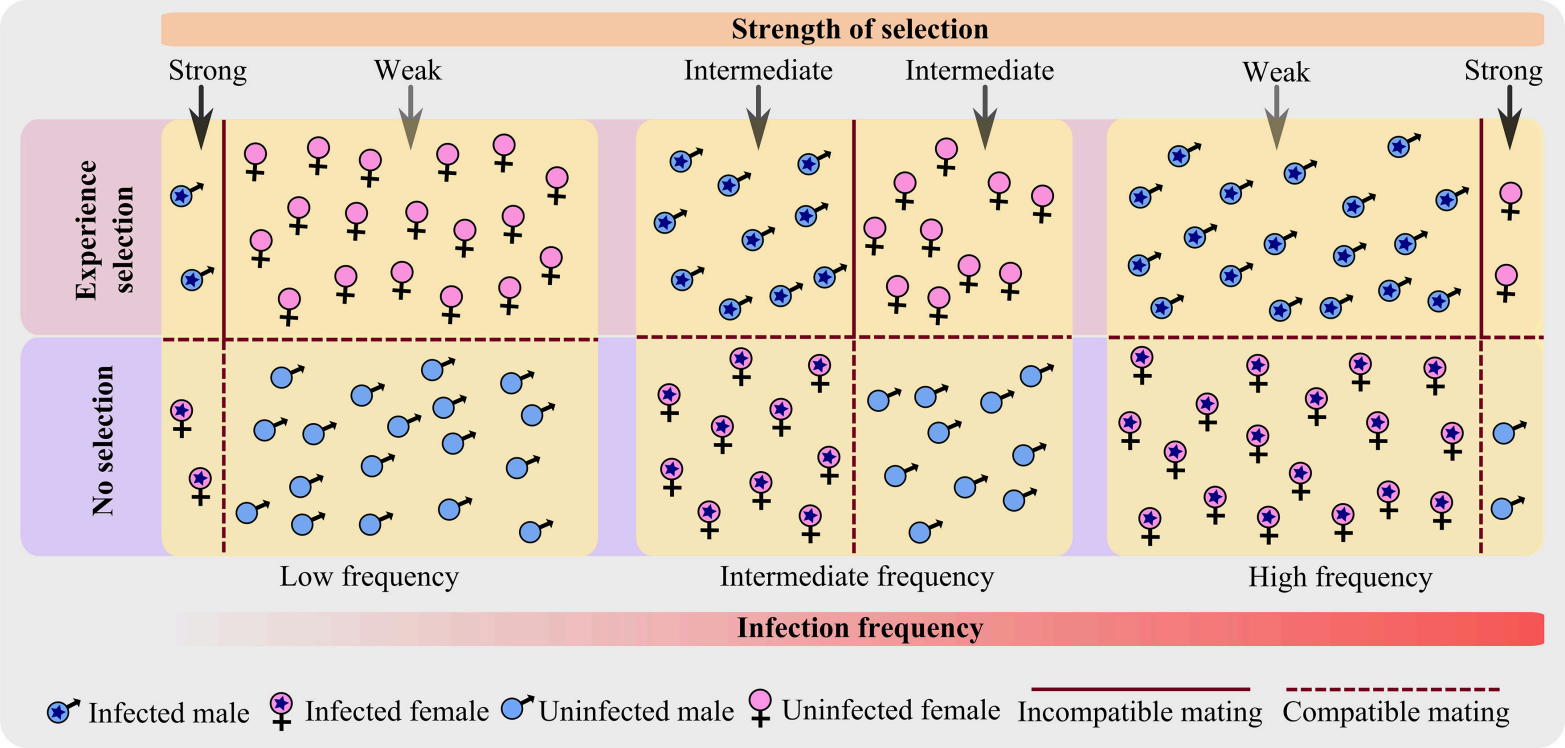
**A schematic representation showing how the strength of selection changes along the infection frequency in a host population infected with CI-inducing bacterium**. The diagram shows the advancement of infection frequency in a host population of equal sex ratio and constant population size; where both male and female are equally susceptible to infection. The host population is categorized into four types based on the sex and infection state. At any particular infection frequency (low, intermediate, or high), only two categories are under selection—infected male and uninfected female. As infection advances, the strength of selection changes from strong to weak for an infected male; and from weak to strong for an uninfected female.

As a result, only infected males and uninfected females are under the influence of selection pressure (Crespigny and Wedell, 2007), rather than the whole population (Figure 1). Even more, with an increase in infection prevalence, the frequency of infected male increases and that of uninfected female decreases; and thus affects the CI frequency in the population which in turn influences the strength of selection.

As the theoretical analyses predict: after the infection frequency transgressed a critical threshold value (~0.08 proportion of the population; Turelli and Hoffmann, 1995), *Wolbachia* spreads rapidly in the host population (Hoffmann et al., 1990; Champion de Crespigny et al., 2005). Hence, I have assumed that the infection prevalence is usually either low or high, and intermediate frequency only exists for short time spans.

### Spatial and temporal variation in fitness cost

Following a *Wolbachia* infestation in a population, before *Wolbachia* reaches the threshold frequency and becomes dominant, the host population comprises a high proportion of uninfected individuals. Therefore, the fitness of an infected male is greatly reduced because of the higher chance that it would mate with an uninfected female, and thus produce no offspring. Even though the frequency of incompatible mating is very low at the population level, the fitness cost for each infected male is very high, and these few individuals experience strong selection for traits that would (i) counteract CI and produce fertile offspring with uninfected females or (ii) enable them to avoid courting or mating with uninfected females. At the same time, the chance that an uninfected female would mate with an infected male (and thus not produce viable offspring) is low. Therefore, while there are many uninfected females that selection could act upon, the force of selection on females is low when *Wolbachia* frequency is low.

When *Wolbachia* prevalence transgresses the threshold frequency, the situation becomes opposite, with a large population of infected males that experience only weak selection, and a small population of uninfected females that experience strong selection. Thus, selection for antagonistic traits is either hampered by small population size to act on or weak force. Moreover, the selection shifts between the infected male and uninfected female as *Wolbachia* becomes dominant; as a result, it is most likely that the antagonistic traits selected for and their fitness value will also change (Turelli, 1994; Correa and Ballard, 2016).

### Ci-antagonism in host

At low infection prevalence, evolution of antagonistic strategies are impeded for infected males, because the number of individuals under selection is very low; so there is less chance that such a strategy would be present among the individuals under selection; and in case it is there, it would be more prone to elimination through drift (Champion de Crespigny et al., 2005). Corroborating this prediction, it is found that in *Wolbachia*-infected *Nasonia vitripennis*, male mating history had no effect on CI induction (Clark et al., 2008). However, in *Drosophila simulans*, multiple mating by infected males increase their reproductive output (Awrahman et al., 2014). Nevertheless, such behavioral strategies could arise because of species-specific reproductive biology rather than an explicit evolution in response to CI (Awrahman et al., 2014).

At high infection prevalence, low frequency of uninfected females reduces the chances for the evolution of antagonistic strategies due to similar reasons as explained above. Or else, at a lower frequency of uninfected females, the preference allele if any may suffer fitness cost due to delayed mating (Champion de Crespigny et al., 2005). In one of the experiments, the uninfected females of *Tetranychus urticae* showed a mating preference for uninfected males in the choice experiment (Vala et al., 2004). However, the evolution of such mating preference is debatable, because such preference does not exist in a panmictic mating scenario (Crespigny and Wedell, 2007).

### At intermediate infection frequency

Theoretical analyses show that an intermediate *Wolbachia* frequency cannot be stable (Champion de Crespigny et al., 2005), because after threshold frequency, the infection rapidly spread to fixation. Field studies, however, reveal the intermediate frequency of infection in certain natural populations (Hoffmann et al., 1990; Werren and Windsor, 2000; Vala et al., 2004; Hughes et al., 2011). Such intermediate frequency has been attributed to stochastic environmental events (e.g., environmental curing), incomplete CI, or imperfect maternal transmission (Hoffmann et al., 1990; Turelli and Hoffmann, 1995; Reynolds and Hoffmann, 2002; Champion de Crespigny et al., 2005). At such an intermediate frequency of infection, for instance, when the infection frequency is 0.5 (Figure 1), the population level incidence of CI (infected males mating uninfected females) is most frequent due to an equal number of both infected males and uninfected females in the population. However, the probability that an infected male will mate with an uninfected female or vice versa (incompatible cross) is almost equal to that of the compatible crosses. Hence, even at intermediate infection frequencies, the selection for any CI antagonistic traits is not very strong, and such intermediate frequencies may exist only for a short period of time. However, these speculations need to be tested with explicit experiments, for instance:

1. Can a host population remain at the intermediate infection frequency for a considerable period? This question addresses the occurrence and consistency of intermediate *Wolbachia* frequency in a population. Systematic release of infected or uninfected individuals to an experimental population of host isofemale lines can be used to test these predictions.
2. What is the cost and benefit of a preference allele in a population that remain at intermediate infection frequency?

## Concluding remarks

Thus, the main thesis is that under the influence of CI, the host population experiences a dynamic scenario where the selection for CI-antagonism is experienced by certain individuals based on their sex and the infection state; and the strength of selection changes depending upon the infection frequency in the population. As *Wolbachia* advances, the selection (i) shifts between infected male and uninfected female and (ii) selection strength is determined by the number of individuals in these categories. As a result, the strength of selection upon each individual and the number of individuals under the selection inversely correlate, which led me to presume that this counteraction diminishes the chance of fixation of any antagonistic variation against CI-phenotype. Nevertheless, I surmise that inclusion of both spatial (e.g., proportion of population under selection) and temporal (e.g., selection shift) components of the population, in studying the fate of any antagonistic variation, would be necessary to understand the evolution of host responses to CI.

### Conflict of interest statement

The author declares that the research was conducted in the absence of any commercial or financial relationships that could be construed as a potential conflict of interest.
